# Insulator Defect Detection Based on ML-YOLOv5 Algorithm

**DOI:** 10.3390/s24010204

**Published:** 2023-12-29

**Authors:** Tong Wang, Yidi Zhai, Yuhang Li, Weihua Wang, Guoyong Ye, Shaobo Jin

**Affiliations:** 1 Henan Key Laboratory of Intelligent Manufacturing of Mechanical Equipment, Zhengzhou University of Light Industry, Zhengzhou 450002, China; 2 College of Mechanical and Electrical Engineering, Zhengzhou University of Light Industry, Zhengzhou 450002, China; 3 China Special Equipment Inspection and Research Institute, Beijing 100029, China

**Keywords:** convolutional neural networks, object detection, feature fusion, attention mechanisms

## Abstract

To address the challenges of balancing accuracy and speed, as well as the parameters and FLOPs in current insulator defect detection, we propose an enhanced insulator defect detection algorithm, ML-YOLOv5, based on the YOLOv5 network. The backbone module incorporates depthwise separable convolution, and the feature fusion C3 module is replaced with the improved C2f_DG module. Furthermore, we enhance the feature pyramid network (MFPN) and employ knowledge distillation using YOLOv5m as the teacher model. Experimental results demonstrate that this approach achieved a 46.9% reduction in parameter count and a 43.0% reduction in FLOPs, while maintaining an FPS of 63.6. It exhibited good accuracy and detection speed on both the CPLID and IDID datasets, making it suitable for real-time inspection of high-altitude insulator defects.

## 1. Introduction

Insulators are essential components of high-voltage transmission lines, providing insulation and mechanical support. Insulator faults are primarily caused by high leakage current and harsh working conditions [1], jeopardizing the stability of transmission lines and posing safety risks. Therefore, regular inspection of insulator faults is of significant importance for ensuring the stability of the electrical power system [2].

The common faults of insulators include flashover and broken. Broken is mainly caused by external forces during transportation, installation, construction, and other processes, leading to injury to the insulator. Flashover is mainly caused by lightning or overvoltage, which can easily result in flashover and breakdown faults, affecting the operation of power systems. Hammer are designed to reduce vibrations caused by wind, preventing conductor vibration. High-voltage overhead line poles are positioned at a considerable height, with large spans. When the conductor is subjected to wind forces, it may experience vibration. Due to repeated vibrations, the conductor undergoes fatigue damage due to periodic bending. Therefore, the daily detection of insulator broken, flashover, and the effectiveness of hammer is of great significance.

Traditional insulator inspection primarily relies on manual methods such as ground patrols, instrument measurements, and pole climbing, which are time-consuming, labor-intensive, and costly. With the widespread adoption of drones, high-altitude inspections are now commonly conducted using unmanned aerial vehicles (UAVs). These UAVs employ algorithms to accurately detect and address insulator defects [3].

Dai et al. [4] improved deep convolutional neural networks (DCNNs), using adaptive strategies to enhance the efficiency of drone inspections. Ghashghaei et al. [5] performed defect detection on insulator images captured by drones using Faster R-CNN, achieving increased accuracy at the cost of significantly increased model complexity. Zhou et al. [6] introduced attention mechanisms and rotation mechanisms into the backbone network and loss functions of Mask R-CNN, employing multi-angle rotations to enhance localization accuracy.

However, given the vast scale of the power grid and the intricate structures between transmission lines in China, the use of UAVs for inspections cannot guarantee accuracy and safety. As a result, object detection algorithms have been introduced and widely applied in UAV-based insulator defect detection [7].

Object detection algorithms can be primarily categorized into two types: single-stage object detection and two-stage detection algorithms. Single-stage object detection directly identifies objects in images and is better suited for real-time requirements. Classical single-stage detection algorithms include the YOLO series [8,9,10] and the SSD series [11,12,13].

In the realm of insulator defect detection, Liu et al. [14] improved the YOLO network by employing multiple-scale detection heads and multi-scale feature fusion to enhance insulator detection accuracy, particularly in complex backgrounds. Sadykova et al. [15] achieved precise insulator localization using YOLOv2 and conducted conditional classification based on factors such as cleanliness, water, and snow conditions. Adou et al. [16] employed the YOLOv3 network for insulator localization and bundle detection. Li et al. [17] proposed an insulator detection method for power grids based on light calibration enhancement and YOLOv5. Feng et al. [18] presented an automatic insulator detection approach using YOLOv5. Han et al. [19] conducted real-time insulator defect detection using YOLO-v3. Wang et al. [20] proposed an insulator detection model, Siamese ID-YOLO. The model enhances semantic information using the Canny operator and further introduces a Siamese network based on the Darknet53 architecture to improve the accuracy of insulator detection. Chen et al. [21] proposed an insulator defect detection method called INSU-YOLO. The approach utilizes ResNet101 to extract features at different levels. It incorporates the feature pyramid network (FPN) with the path aggregation network (PAN) from YOLOv4 for feature transfer, employing a bottom-up structure. Finally, a detector is used for classification. Liu et al. [22] proposed a cross-stage partial dense YOLO (CSPD-YOLO) model based on YOLO-v3 and a cross-stage partial network. Han et al. [23] proposed a cascaded model for detecting multiple insulator faults in aerial images. Firstly, they introduced a new spatial pyramid pooling (SPP) improved network structure for detecting insulator strings. Secondly, the YOLOv3-tiny network was employed to detect insulator missing faults. Souza et al. [24] proposed a hybrid approach that combines the optimal insulator detection model YOLOv5x with the optimal insulator state classification ResNet-18 structure, providing a method superior to the standard model.

To reduce system losses and computational complexity, lightweight networks are commonly adopted for insulator defect detection. Xu et al. [25] improved YOLOv1 by utilizing the Mobilenet-V4 backbone network and introduced depthwise separable convolutions to reduce feature redundancy. Liu et al. [26] proposed an enhanced YOLOv3 model based on SPPNet [27] and multi-scale predictions to improve insulator detection accuracy under different aerial imaging backgrounds. Lan et al. [28] introduced Ghost lightweight modules and attention mechanisms into YOLOv5 for insulator defect detection. Zhang et al. [29] proposed an insulator defect detection algorithm based on YOLOv7. The approach incorporates ECA, utilizes PConv in the backbone network, and employs Normalized Wasserstein Distance (NWD) to prevent feature loss. Guo et al. [30] replaced the YOLOv5 backbone network with Transformer-CSP to reduce computational complexity. Additionally, they introduced an insulator defect segmentation head network for defect segmentation. Chen et al. [31] proposed an enhanced insulator defect recognition algorithm, Insu-YOLO, based on the latest YOLOv8 network. By introducing the GSConv module and employing the lightweight CARAFE structure in the neck network, the accuracy of detecting small targets was improved. Shuang et al. [32] proposed an enhanced network, YOLOv4++, based on the improvements made to YOLOv4. The backbone network utilizes MobileNetv1 with depthwise separable convolution, and the model’s effectiveness is further enhanced through improvements in the loss functions. The aforementioned deep learning-based methods outperform traditional algorithms. However, these methods typically demand substantial computational resources, especially in scenarios with complex backgrounds and a small proportion of defects. This poses significant challenges for real-time applications and environments with limited resources. Therefore, there is an urgent need to address the trade-off between detection speed and accuracy, reducing computational requirements to meet the efficiency and accuracy demands of daily inspections in the power industry.

To address the challenges of balancing the speed and accuracy in insulator defect detection, as well as the FLOPs and parameter burdens, we propose an enhanced network, ML-YOLOv5, based on YOLOv5. The main contributions of this work are as follows:

1. Introducing depthwise separable convolution (DSC) into the backbone network of the YOLOv5 model to reduce feature redundancy. In the feature fusion component, an improved C2f_DG is employed to construct a lightweight structure, significantly reducing the model’s parameter count and computational complexity while maintaining accuracy.
2. The enhanced feature pyramid, designated as MFPN (modified feature pyramid network), strengthens the original FPN structure by increasing the number of concatenate layers from two to three. It incorporates shallow-level feature maps and substitutes the C3 module corresponding to the shallow-level features of the backbone network with C2f.
3. Utilizing the sibling deep network YOLOv5m as the teacher network, knowledge distillation [33] was applied to train the improved lightweight network. This approach significantly enhances detection accuracy while keeping the model size unchanged.
4. This method exhibits high accuracy and detection speed on two open-source datasets, ensuring real-time performance while maintaining excellent detection precision. It can be employed for real-time detection of high-altitude insulator defects.

## 2. Insulator Defect Detection Algorithm

### 2.1. YOLOv5 Algorithm

**Figure 1:**
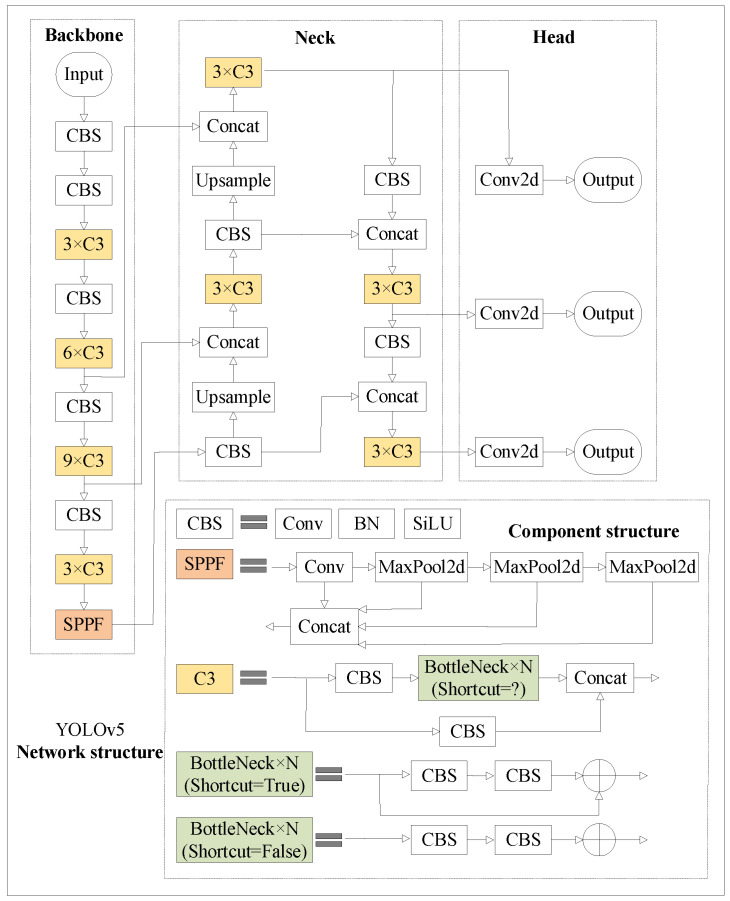
YOLOv5 model structure diagram.

YOLOv5 offers five models of varying scales: n, s, m, l, and x, each with distinct depths and widths. To achieve efficient detection of small insulator defects, this study adopts the simplified YOLOv5s architecture. The original YOLOv5 network architecture is illustrated in Figure 1.

YOLOv5 consists of three modules: feature extraction (backbone), neck, and head. The backbone includes the CBS (Conv-BatchNorm-SiLU), C3, and SPPF modules, where CBS is employed for feature extraction, C3 deepens the network to enhance feature extraction, and SPPF is used for multi-scale feature fusion. The neck module comprises FPN and PAN, which combine deep- and shallow-level semantic features. The head is designed with multiple detection layers for different sizes, and the detection results are obtained through loss calculation and non-maximum suppression.

### 2.2. DWconv and Ghostconv

In this work, depthwise separable convolutions and Ghost Models are introduced in the backbone network and the enhancement module to replace conventional convolutions, reducing feature redundancy.

**Figure 2:**
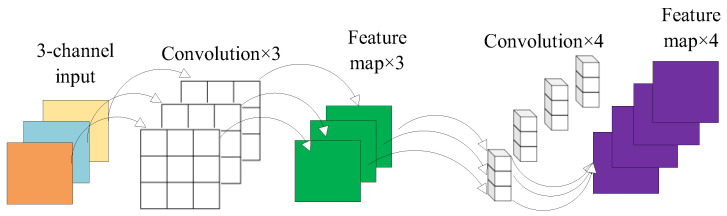
Depthwise separable convolution structure.

As shown in Figure 2, depthwise separable convolution divides feature extraction into depthwise convolution and pointwise convolution, targeting the extraction of spatial and channel features from the input data. In depthwise convolution, each kernel is responsible for one channel, and the number of generated feature maps matches the number of input data channels, with each channel corresponding to an output feature map.

As a result, channelwise convolution alters the size of feature maps without changing the number of feature map channels. Since depthwise convolution independently convolves each channel of the input data, subsequent pointwise convolution is used to reassemble these independent feature maps, aiding in the extraction of channel features from the input data. In contrast to depthwise convolution, it does not change the size of feature maps but only alters the number of channels.

**Figure 3:**
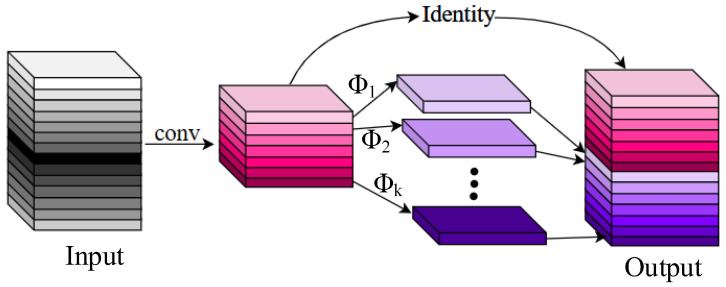
The structure of the GSConv.

As shown in Figure 3, the Ghost Model obtains feature maps in two steps. First, it uses half of the convolutions to acquire the intrinsic feature maps, reducing the parameter count by half. Then, it employs cheap operations denoted by Φ to subject the feature maps to depthwise separable convolution individually, resulting in the Ghost feature maps. Finally, the intrinsic feature maps and Ghost feature maps are identity-concatenated to yield the Output.

### 2.3. C2f and Its Improved Modules

**Figure 4:**
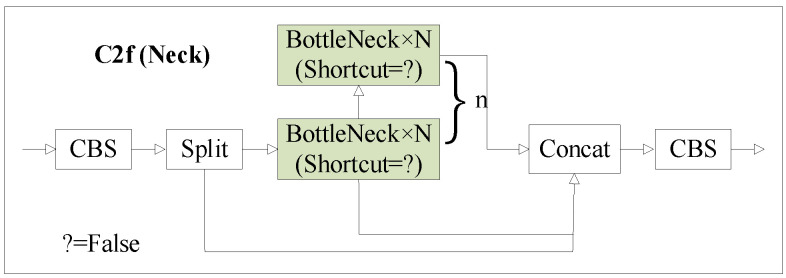
The structure of the C2f.

In this study, the bottleneck module integrates the C2f module from YOLOv8 with the option shortcut = False, indicating the absence of residual connections. This design choice aims to reduce the model’s parameter count and computational load, as illustrated in Figure 4. This module draws inspiration from the ELAN architecture of YOLOv8 and the C3 module of YOLOv5, introducing cross-layer branching and gradient flow information to enhance robust feature representation.

**Figure 5:**
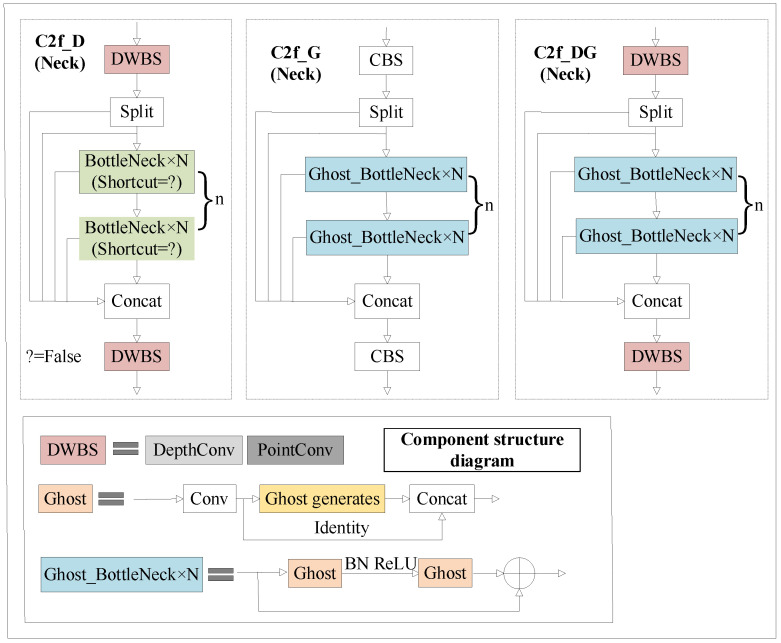
C2f improves modules C2f_D, C2f_G, and C2f_DG.

In this paper, we have designed three modified C3 modules, namely C2f_D, C2f_G, and C2f_DG, to replace the original feature fusion C3 module in YOLOv5. The improved C2f structure is depicted in Figure 5, Among them, shortcut = False means that there is no residual connection.

C2f_D is an enhancement of the C2f module in YOLOv8, inheriting its advantages. It employs depthwise separable convolution (DWConv) instead of conventional convolution, forming the DWBS module (DWConv-BatchNorm-SiLU). This module allows the independent extraction of spatial and channel features from input data, effectively reducing feature redundancy. C2f_G replaces the bottleneck in the original C2f with a Ghost Bottleneck, which incorporates cross-layer fusion and gradient truncation mechanisms to facilitate cross-level feature extraction, reduce model size, and enhance training performance. The structure of the Ghost Bottleneck consists of two Ghost modules. The role of the first Ghost module is to increase the channel number of the input feature map, providing extension for subsequent operations. The role of the second Ghost module is to reduce the channel number of the output feature map to match the network’s diameter structure, and it facilitates information transmission between the two Ghost modules through the diameter structure. Additionally, from the diagram, it can be observed that the difference between the two Ghost modules lies in the fact that the first Ghost module is followed by the ReLU activation function, while subsequent layers are all subjected to batch normalization. This structural approach not only effectively reduces model parameters and computational complexity but also optimizes feature maps through the Ghost modules, thereby enhancing the model’s detection efficiency. C2f_DG combines the improvement strategies of both C2f_D and C2f_G, achieving a balance between reducing parameters and maintaining network performance.

### 2.4. Improved Feature Pyramid MFPN

**Figure 6:**
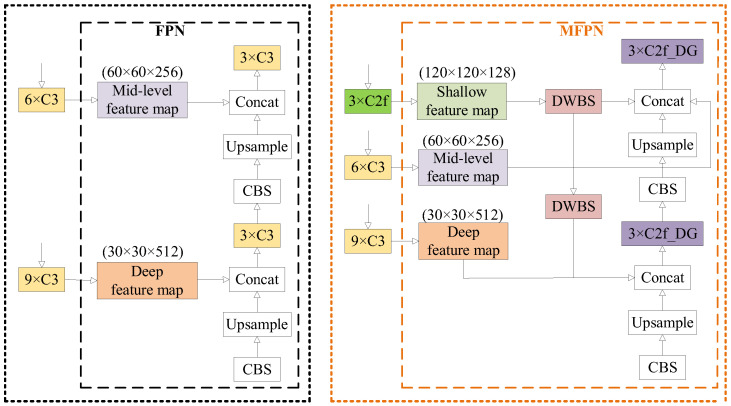
Comparison of FPN and MFPN modules before and after improvement.

In order to enrich feature information without introducing additional parameters, we expanded the number of cascaded feature maps in the feature pyramid network (FPN) structure from the original two layers to three layers. Furthermore, the C3 module of the backbone network corresponding to the added shallow feature maps was replaced with C2f. We designed an enhanced multi-scale feature pyramid network (MFPN) for this purpose. Comparison images before and after the improvements to the FPN and MFPN are shown in Figure 6.

As depicted in the figure above, the FPN structure involves concatenating only the upsampled feature maps from the lower layers with the deep- or mid-level feature maps of the backbone network. In contrast, the improved MFPN, building upon the FPN framework, introduces shallow-level feature maps, increasing the number of concatenated layers from two to three. To ensure consistency in feature map sizes, depthwise separable convolutions are introduced to expand the number of channels. Shallow networks contain more target-specific information, enabling better expression of feature map positional information. However, they have a smaller receptive field and weaker semantic expressive capability. In contrast, deep networks possess stronger expressive capabilities but have the opposite characteristics. The improved feature pyramid fusion incorporates richer information from deep, intermediate, and shallow layers, facilitating the comprehensive identification of multi-defect insulator images and preventing the loss of information regarding small target defects.

### 2.5. Knowledge Distillation

**Figure 7:**
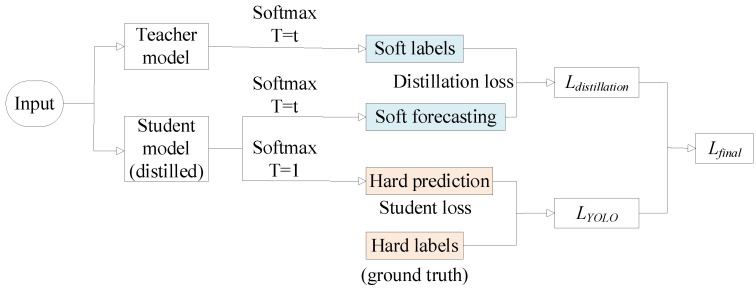
Knowledge distillation flow chart.

Knowledge distillation is a technique that leverages knowledge transfer to train a lightweight student network from a larger teacher network. In this study, we apply the knowledge distillation method proposed by Mehta et al. [34] to distill the improved model. The knowledge distillation process is illustrated in Figure 7.

As shown in the figure above, the output values of the Softmax function, with an added variable T, are used as soft targets to prevent minimal contributions to the loss function when the probability distribution has low entropy. The Softmax function is defined by the following equation:

(1) $$q_{i}=\frac{\exp(z_{i}/T)}{\sum_{j}\exp(z_{j}/T)}$$

In the equation, q_i_ represents the true probability, z is a vector, and z_i_ and z_j_ are elements of the vector. T represents the temperature, where higher T values result in a larger entropy in the Softmax distribution, increasing the focus on training negative labels.

The overall loss consists of algorithmic and knowledge distillation losses, with λ_D_ used to balance these two types of losses. The total loss function is expressed as shown in Equation (2).

(2) $$L_{\mathrm{final}}=L_{YOLO}+\lambda_{D}.L_{Distillation}$$

In this study, we made improvements to the YOLOv5s, a lightweight network model, and YOLOv5m, a deeper network model, as follows:

(1) Integrated depthwise separable convolutions into the backbone.
(2) Replaced the C3 module in the neck with an enhanced C2f_DG.
(3) Replaced the feature pyramid network (FPN) with the modified feature pyramid network (MFPN).

The improved models based on YOLOv5s and YOLOv5m are, respectively, named M-YOLOv5s and M-YOLOv5m. We employed M-YOLOv5m as a teacher network to train the student model M-YOLOv5s. Through knowledge distillation, we improved model accuracy without adding extra parameters.

### 2.6. Improved ML-YOLOv5 Algorithm

**Figure 8:**
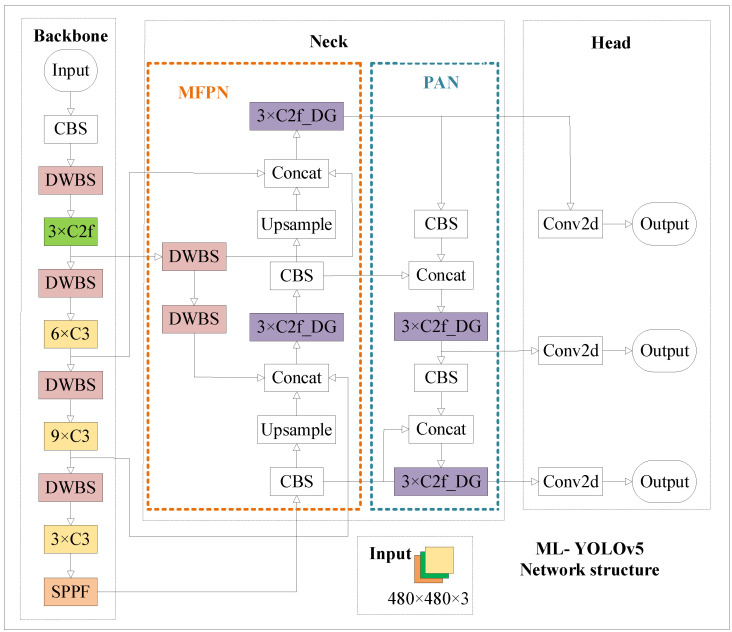
Improved ML-YOLOv5 model structure diagram.

In response to the current challenges of low insulator defect detection accuracy and prolonged computation times, this paper introduces an enhanced insulator defect detection model, ML-YOLOv5, based on YOLOv5s. The architecture of the ML-YOLOv5 network is illustrated in Figure 8, and it incorporates the following improvements:

(1) The backbone integrates depthwise separable convolution;
(2) The C3 module in the neck is replaced with the improved C2f_DG;
(3) Enhancement of the feature pyramid network (MFPN);
(4) Knowledge distillation.

## 3. Experiment

### 3.1. Data Preprocessing

(1) The Chinese Power Line Insulator Dataset (CPLID) [35] comprises a total of 848 aerial images of composite insulators, divided into two categories: 600 images of normal insulators and 248 images of damaged insulators. The dataset was randomly split into training, testing, and validation sets in a 7:2:1 ratio. Manual annotations of the images were performed using LabelImg, and the label distribution is summarized in Table 1.

(2) Lewis et al. [36] collected and curated the Insulator Defect Image Dataset (IDID), which comprises 487 original images classified into four categories: insulator strings, damaged insulators, flashover insulators, and hammer. The dataset was randomly partitioned into training, testing, and validation sets in a 7:2:1 ratio. Data augmentation techniques such as flipping, mirroring, and random cropping were applied to augment the dataset, enhancing model generalization. Ultimately, a total of 4600 images were obtained, and manual annotation was performed using LabelImg. The label distribution is summarized in Table 1.

### 3.2. Experimental Environment and Evaluation Indicators

#### 3.2.1. Experiment Platform

The experiments were conducted using the PyTorch deep learning framework on Windows 11, with an Intel^®^ Core™ i5-12500H 2.50 GHz CPU and an NVIDIA GeForce RTX 3050 Laptop GPU with 4 GB of RAM. During training, the input image size was set to 480 × 480, and a batch size of 16 was used. The optimization was performed using SGD with a momentum of 0.937, and the learning rate was scheduled to decrease from the initial value of 0.01 to 0.001 using cosine annealing.

#### 3.2.2. Evaluation Indicators

To assess the performance of the insulator defect detection algorithm, several widely used evaluation metrics were employed, including precision (P), recall (R), average precision (AP), and mean average precision (mAP0.5). The definitions of these metrics are provided below:

(3) $$P=\frac{TP}{TP+FP}$$

(4) $$R=\frac{TP}{TP+FN}$$

(5) $$AP=\int_{0}^{1}P(R)dR$$

(6) $$mAP=\frac{\sum_{i=1}^{N}AP(i)}{N}$$

In Equations (3)–(6), TP represents the number of correctly predicted insulator defect classes, FP represents the number of incorrectly predicted negative samples, FN represents the number of undetected insulator defects, AP denotes the integral of precision values at different recall rates, N represents the number of label categories, and mAP represents the average AP across different categories.

Parameters and FLOPs are utilized as metrics for assessing the model’s complexity, while frames per second (FPS) reflects the model’s detection speed.

### 3.3. Comparative Experiment

To validate the performance of the improved ML-YOLOv5 algorithm for insulator defect detection, we compared it with other YOLO algorithms within the same series using the CPLID and IDID datasets. Additionally, we replicated the work of Zhang et al. [37], where they introduced the Ghost module and a small object detection layer based on YOLOv5, referred to as YOLOv5-Gh, for small object detection on insulators. At the same time, we reproduced the improved BC-YOLO network proposed by Bao et al. [38]. We integrated the CA attention mechanism into the backbone module and introduced Bi-FPN [39] in the neck module to replace the original PANet, thereby enhancing the network’s capability for detecting small objects. Ding et al. [40] proposed a novel model, GC-YOLO, based on the improvement of YOLOv5s. GC-YOLO integrates the Ghost convolution module into the backbone network, adds the CA attention mechanism, introduces the EVCBlock module in the neck layer, and includes an additional small object detection head in the detection layer. Luan et al. [41] introduced MI-YOLO, an improved network based on YOLOv5, featuring a skip connection module with down-sampling in the backbone network, a neck layer with a spatial pyramid dilated convolution module, and the addition of a novel serial-parallel spatial-channel attention module. The evaluation metrics included precision (P), recall (R), mAP0.5, model parameters, FLOPs (Floating-Point Operations Per Second), and frames per second (FPS). The comparative experimental results are presented in Table 2.

As shown in the table above, in terms of mean average precision at (mAP0.5), ML-YOLOv5 exhibits the best performance, achieving 97.0% and 82.8% on the CPLID and IDID datasets, respectively. This performance is notably superior to that of similar lightweight algorithms of the same category. Compared to the latest model YOLOv8s, it demonstrates significant advantages in both accuracy and computational efficiency. The algorithm also maintains a high FPS of 63.6, only slightly lower than the original YOLOv5, ensuring real-time processing. Furthermore, the improved algorithm exhibits reduced model complexity, with parameters totaling 3.73 M and FLOPs of 9.0, nearly halving those of YOLOv5. In summary, the algorithm proposed in this paper effectively balances the challenges of detection accuracy and speed, reduces model complexity, and meets the real-time requirements of power line inspection.

**Figure 9:**
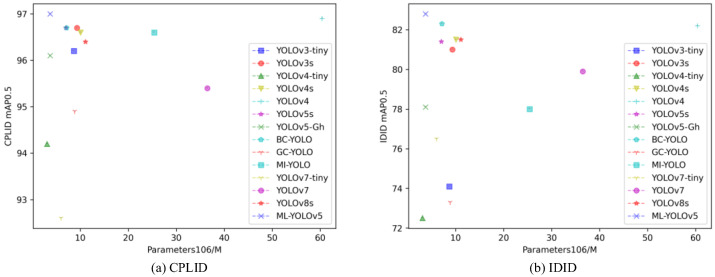
Illustration of the relationship between mAP0.5 and model parameter count for different models.

The relationship between the model parameters and mAP0.5 of the compared models is depicted in Figure 9. The x-axis represents the model parameters, and the y-axis represents the detection performance indicator mAP0.5. In the context of insulator defect detection tasks addressed in this paper, models positioned closer to the upper-left corner are relatively superior. As shown in Figure 9a for the CPLID dataset and Figure 9b for the IDID dataset, compared to other models, YOLOv4-tiny, YOLOv5-GH, and ML-YOLOv5 have smaller model parameters, with ML-YOLOv5 exhibiting the highest detection accuracy. In comparison to all the considered models, ML-YOLOv5 strikes a balance between detection performance and the number of model parameters, making it more suitable for insulator defect detection tasks.

### 3.4. Ablation Experiment

To assess the impact of various improvement modules on network performance, validation was conducted on the IDID test dataset. The ablation results are presented in Table 3, where “√” indicates the inclusion of the respective improvement point.

Model A represents the original YOLOv5s network architecture. Model B incorporates depthwise separable convolution (DSC) into the main structure of Model A. Model C replaces the C3 module in the neck structure of Model A with the improved C2f_DG module. Model D enhances the feature pyramid in Model A using MFPN. Model E, built upon Model B, substitutes the C3 module in the neck module with the improved C2f_DG module. Model F, an extension of Model E, improves the feature pyramid with MFPN. Finally, Model G applies knowledge distillation on top of Model F.

Table 3 presents the results of ablation experiments, demonstrating that the introduction of DSC and the improved C2f_DG significantly reduce both model parameters and computational complexity, resulting in a 0.5% decrease in mAP0.5, aligning with the lightweight design principles. Subsequently, the incorporation of the enhanced feature pyramid MFPN improves model accuracy. Finally, knowledge distillation using the deep network YOLOv5m as the teacher model significantly enhances detection accuracy without altering the model size.

Regarding the selection of the C2f_DG module, we conducted ablation experiments by comparing the regular convolution improvement of C2f_D, which only enhances the C2f with ordinary convolution, and C2f_G, which enhances C2f with bottleneck. The results of the ablation experiments are shown in Table 4, where ‘√’ indicates the inclusion of the corresponding improvement points.

As shown in the table above, replacing the C3 module of the original YOLOv5s with C2f_DG yields the best results in terms of parameters, computations, and FPS performance. It exhibits a slight decrease in accuracy but meets the requirements for lightweight design, providing further evidence of the feasibility of the proposed improvement. Table 5 presents a comparative analysis of experimental results before and after knowledge distillation. In this study, the improved models, M-YOLOv5s and M-YOLOv5m, were employed as the student and teacher networks for knowledge distillation. Prior to knowledge distillation, the M-YOLOv5s network model, which is an enhancement of the baseline model YOLOv5s, achieved an mAP0.5 value of 82.1% on the evaluation metrics. Subsequently, when the deep network model YOLOv5m was used for knowledge distillation as the teacher network, the final model demonstrated improvements in mAP0.5 values of 1.4% compared to YOLOv5s. These improvements were achieved while reducing the number of parameters and FLOPs by 46.9% and 43.0%, resulting in a detection speed of 63.6 FPS.

The above data demonstrates the effectiveness of the proposed improvement method, which achieves a lightweight network and improved prediction accuracy while ensuring real-time on-site detection.

### 3.5. Comparison of Test Results

To validate the effectiveness of the improved algorithm, tests were conducted on the YOLOv5 network models before and after improvement using the CPLID and IDID datasets. The comparison results for mAP across different labels are presented in Table 6.

As demonstrated in Table 6, the average precision of insulator strings in the CPLID dataset has improved by 0.6%. In the IDID dataset, the average precision values for insulator strings, damaged insulators, and flashovers have improved by 0.3%, 4.5%, and 1.3%, respectively. Due to factors such as complex backgrounds and small target defects, issues like false positives and false negatives are prone to occur.

The confusion matrix is a specific two-dimensional matrix where rows and columns represent actual and predicted defect categories, respectively. The values on the diagonal represent the proportion of correct predictions, with higher diagonal values and darker colors indicating better prediction performance. To assess the performance of the improved model in detecting multi-label defects, the confusion matrices before and after improvement are compared on the CPLID and IDID insulator datasets.

**Figure 10:**
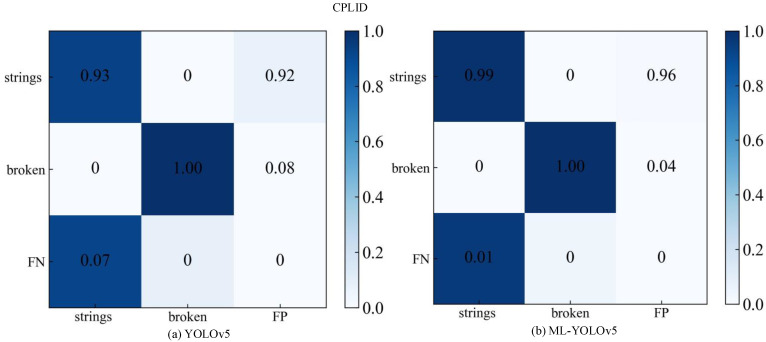
Confusion matrix of the original model and the improved model of the CPLID dataset.

The comparison of confusion matrices for the CPLID dataset is illustrated in Figure 10. Figure 10a presents the confusion matrix results for the original YOLOv5. In the first row and first column, the probability of correctly predicting the insulator string is 93%. In the second row and second column, the probability of predicting insulator damage is 100%. Figure 10b displays the confusion matrix results after improvement using ML-YOLOv5. It can be observed that in the first row and first column, the probability of correctly predicting the insulator string increases to 99%. In the second row and second column, the probability of predicting insulator damage remains at 100%.

After improvement, the ML-YOLOv5 model exhibited a 6% increase in the probability of correctly predicting the insulator string, validating the enhanced performance of the model.

**Figure 11:**
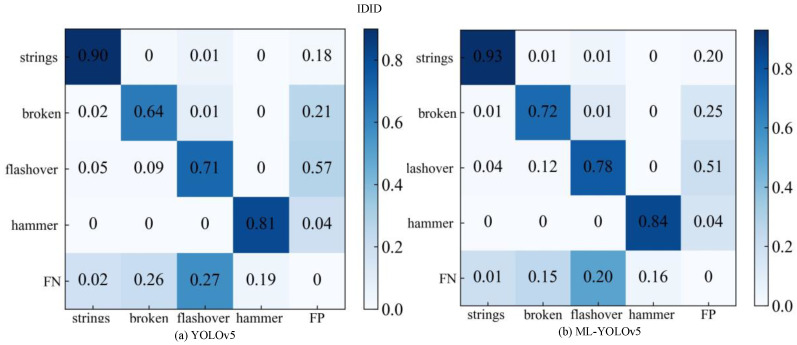
Confusion matrix of the original model and the improved model of the IDID dataset.

The comparison of confusion matrices for the IDID dataset is illustrated in Figure 10. The matrix comparison is illustrated in Figure 11. Figure 11a displays the original YOLOv5 confusion matrix results. In the first row and first column, the probability of correctly predicting insulator strings is 90%. In the second row and second column, the probability of predicting insulator damage is 64%. In the third row and third column, the probability of correctly predicting insulator flashing is 71%. In the fourth row and fourth column, the probability of predicting insulator shock absorbers is 81%.

Figure 11b shows the confusion matrix results after improvement with ML-YOLOv5. It can be observed that in the first row and first column, the probability of correctly predicting insulator strings has increased to 93%. In the second row and second column, the probability of predicting insulator damage has increased to 72%. In the third row and third column, the probability of correctly predicting insulator flashing has increased to 78%. In the fourth row and fourth column, the probability of predicting insulator shock absorbers has increased to 84%.

After improvement, the ML-YOLOv5 model exhibits an improvement in the probabilities of correctly predicting insulator strings, insulator damage, insulator flashing, and insulator shock absorbers by 3%, 8%, 7%, and 3%, respectively.

**Figure 12:**
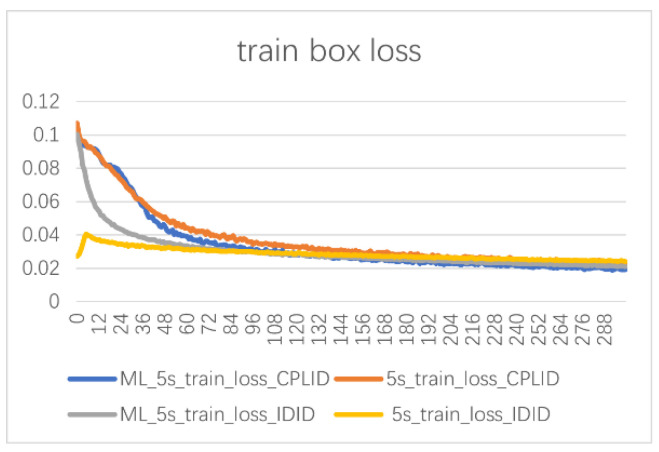
Loss curves of bounding box regression during training for different models.

The loss function curves for different training epochs are shown in Figure 12. Comparing the open-source datasets CPLID and IDID, the improved model demonstrates a faster reduction in loss during the early stages of training compared to the YOLOv5s baseline model. At the end of training, the loss value of ML-YOLOv5 is lower than that of the original model, indicating that the improved network can optimize feature extraction capabilities and effectively enhance the ability to identify minor defects, thereby improving detection performance.

**Figure 13:**
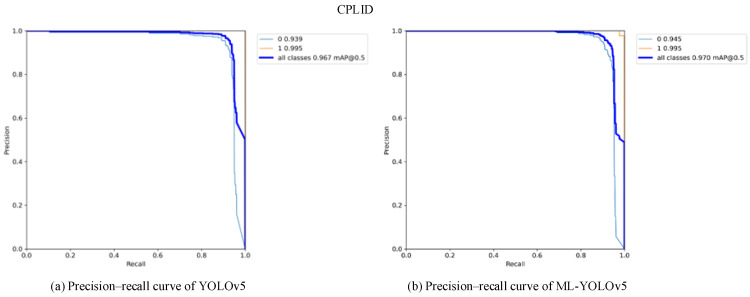
Comparison of precision–recall curves before and after improvement on the CPLID dataset.

**Figure 14:**
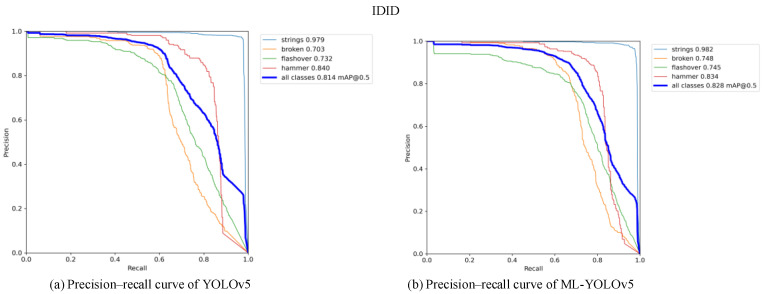
Comparison of precision-recall curves before and after improvement on the IDID data set.

Figure 13 and Figure 14 present the comparison of precision–recall (PR) curves validated on the CPLID and IDID datasets, respectively. The x-axis represents recall, and the y-axis represents precision. The area surrounded by the PR curve and the axes indicates the average precision (AP) value. As shown in Figure 13b, the mAP0.5 of the improved model on the CPLID dataset increased by 0.3%, and in Figure 14b, the mAP0.5 of the improved model on the IDID dataset increased by 1.4%. Across different datasets, there is a noticeable improvement in the average AP of label categories in the ML-YOLOv5 model.

**Figure 15:**
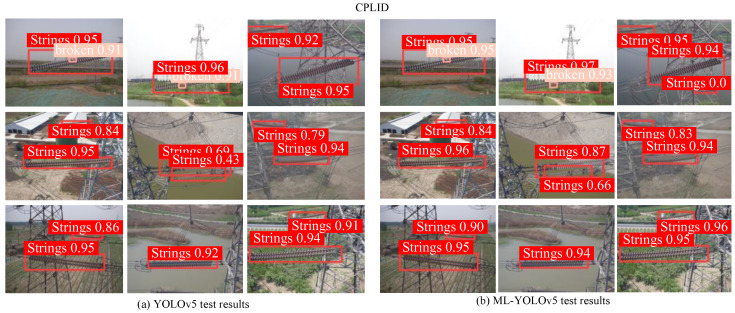
Comparison of detection results of CPLID dataset.

On the CPLID insulator dataset, the detection results before and after improvement are shown in Figure 15, with labels categorized as ‘strings’ and ‘broken’. Figure 15a presents the original YOLOv5 detection results, while Figure 15b displays the results after YOLOv5 improvement. From the figures, it can be observed that there were no false positives or false negatives before and after improvement. However, the improved ML-YOLOv5 exhibited a significant improvement in precision, with detection accuracy reaching as high as 100% for some defect images. This indicates the effectiveness and feasibility of the enhanced lightweight model, ML-YOLOv5.

**Figure 16:**
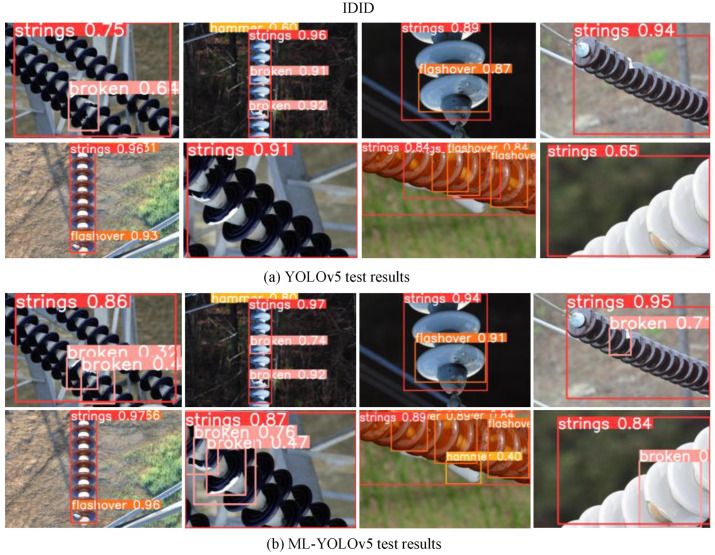
Comparison of detection results of IDID dataset.

On the IDID insulator dataset, the defect detection results before and after improvement are shown in Figure 16. Figure 16a displays the original YOLOv5 detection results, where the leftmost image shows the failure to recognize the ‘hammer’, and false detection of ‘hammer,’ and ‘broken’ is not identified. This is mainly due to the complexity of the background, the small proportion of defects such as damage, and the overall low accuracy. In contrast, Figure 16b shows the results after the improvement of YOLOv5, with a significant improvement in the detection of small target defects, an overall increase in accuracy, and a reduction in the false-negative rate.

In summary, the algorithm improvements presented in this paper have enhanced both accuracy and model lightweightness, ensuring real-time processing requirements.

## 4. Discussion

The proposed ML-YOLOv5 model in this paper is primarily designed for recognizing insulator defects, demonstrating potential applications in the identification of multiple labels and small targets.

The decision to base our algorithm primarily on YOLOv5, rather than the latest version YOLOv8, is motivated by several considerations. Firstly, YOLOv8 is relatively new and may lack widespread adoption and comprehensive evaluation. In contrast, YOLOv5, as a mature, stable, and widely used version, stands out, providing a reliable foundation for our research. Secondly, our focus is on providing a practical solution for insulator defect detection, striking a balance between accuracy, computational efficiency, and real-time performance. Through our comparative experiments and analysis, particularly detailed in Table 2 and Figure 9, YOLOv5 has demonstrated outstanding performance, making it an ideal choice for our specific application requirements.

Despite making progress in this research, certain challenges persist. In comparison to the baseline YOLOv5s model, the improved model shows enhancements in metrics such as mAP0.5, achieving a balance between real-time performance, lightweight modifications, and increased accuracy. However, instances of false negatives and false positives still exist, indicating the need for more diverse datasets to enhance the training effectiveness of the model. Subsequent efforts could involve capturing images from different angles or acquiring more complex background images to further enhance the model’s accuracy and generalization.

In this study, our aim is to achieve lightweight improvements and enhance accuracy while ensuring real-time performance of the model. However, due to the complex backgrounds of insulator defect images and the relatively small proportion of small target defects, there is still room for improvement in accuracy. Therefore, our future work will consider a two-step approach for insulator string detection and defect recognition. Initially, we will use object detection algorithms to identify and crop images of insulator strings. Subsequently, these images containing insulator defect images will serve as a new dataset for recognizing or segmenting small target defects, eliminating background interference, and enhancing detection accuracy.

In addition, we have observed outstanding performance in other advanced models. For instance, Liu et al. [42] proposed a cascaded YOLO model for defect detection. Initially, it recognizes insulators based on the YOLO model, and subsequently, the identified insulator images are processed using the YOLOv4-tiny model for insulator defect detection. Zhang et al. [43] introduced an insulator defect detection method based on YOLO and SPP-Net. YOLOv5s was used to train original samples, and the classification network was fine-tuned with cropped samples for model cascading. After insulator localization and cropping, YOLOv5s sends the images to the classification network for defect detection. Xiong et al. [44] presented a deep cascaded multitask framework, YOLO Unet, for a fully automatic neural network framework for lung nodule detection and segmentation. These works inspire us, and in the future, we will strive to explore and enhance more models to achieve superior performance.

## 5. Conclusions

In this paper, we improve upon YOLOv5 to address the difficulties in efficiently balancing the detection of the performance and speed of high-altitude insulators, as well as the challenges related to parameters and FLOPs. Specifically:

(1) Introducing the DSC module into the backbone;
(2) Replacing the C3 module in the neck with the C2f_DG module;
(3) Enhancing the feature pyramid using MFPN;
(4) Conducting knowledge distillation on the improved lightweight model.

The experimental results demonstrate that the improved ML-YOLOv5 network presented in this study achieved reductions in both parameter count and FLOPs, down to 3.73 M and 9.0, respectively, compared to YOLOv5. The frame rate per second (FPS) reached 63.6. This method exhibited excellent accuracy and detection speed on both the CPLID and IDID datasets, and exhibited improvements in mAP0.5 values of 0.3% and 1.4% compared to YOLOv5s, rendering it suitable for the real-time inspection of high-altitude insulator defects. While the current detection models demonstrate the capability to accurately detect defects, they face challenges when it comes to assessing the precise severity of multiple defects, particularly in improving the recognition of small target defects. In future research, the idea may be considered to combine object detection and semantic segmentation to further enhance the accuracy of small object defect detection. This has significant implications and promising prospects for the field of unmanned aerial vehicle (UAV) fault inspections.

## Figures and Tables

**Table 1:** Label number distribution of CPLID and IDID datasets.

Label Name	CPLID Label Number	IDID Label Number
strings	848	5155
broken	248	3095
flashover	\	5736
hammer	\	1182

**Table 2:** Comparative experimental results of different models.

	CPLID			IDID					
Method	P/%	R/%	mAP0.5/%	P/%	R/%	mAP0.5/%	FPS/s	Parameters 10^6^/M	FLOPs/G
YOLOv3-tiny	96.6	95.6	96.2	80.7	67.5	74.1	63.4	8.67	12.9
YOLOv3s	97.7	94.2	96.7	87.5	75.8	81	61.4	9.31	23.1
YOLOv4-tiny	93.4	92.9	94.2	76	67.2	72.5	63.1	3.07	6.3
YOLOv4s	97.6	96	96.6	90.3	75.4	81.5	42.7	10.07	21.4
YOLOv4	96.6	96.2	96.9	86.4	77.1	82.2	16.2	60.4	130.7
YOLOv5s	97.7	95.4	96.7	87.7	74.2	81.4	64.1	7.02	15.8
YOLOv5-Gh [38]	96.9	94.2	96.1	86.4	71.4	78.1	47.1	3.74	8.2
BC-YOLO [39]	96.8	94.6	96.7	90.7	73	82.3	57.6	7.16	16.6
GC-YOLO [40]	94.7	90.9	94.9	79.9	65	73.3	57.4	8.83	27.8
MI-YOLO [41]	97.9	93.8	96.6	84.4	70.8	78	34.1	25.4	73.1
YOLOv7-tiny	92.2	93.5	92.6	86.3	72	76.5	62.5	6.02	13
YOLOv7	78.3	89.6	95.4	91.7	78.6	79.9	30.5	36.5	103.2
YOLOv8s	96.3	92.5	96.4	84.5	74.1	81.5	56.9	11.1	28.4
ML-YOLOv5	96.5	94.7	97	86.1	77.7	82.8	63.6	3.73	9

**Table 3:** Ablation experimental results of different improved modules.

Method	DSC	C2f_DG	MFPN	KD	P/%	R/%	mAP0.5/%	Parameters 10^6^/M	FLOPs/G	FPS/s
A					87.7	74.2	81.4	7.02	15.8	64.1
B	√				87.6	73.3	81.3	5.46	12.1	59.8
C		√			86	75.6	81	5.16	11.7	59.7
D			√		87.4	74.7	82.2	7.69	19.5	59.3
E	√	√			85.4	73.9	80.9	3.6	8	67.3
F	√	√	√		85	76.6	82.1	3.73	9	63.6
G	√	√	√	√	86.1	77.7	82.8	3.73	9	63.6

**Table 4:** C2f_DG, the results of the ablation experiment of the module.

Method	C2f_D	C2f_G	C2f_DG	P/%	R/%	mAP0.5/%	Parameters 10^6^/M	FLOPs/G	FPS/s
A				87.7	74.2	81.4	7.02	15.8	64.1
B	√			86.6	75.2	81.1	6.3	15.4	54.7
C		√		87.6	74.4	81.4	6.3	14.2	57.6
D			√	86	75.6	81	5.16	11.7	59.7

**Table 5:** Knowledge distillation comparative experiment.

Method	P/%	R/%	mAP0.5/%	FPS/%	Parameters 10^6^/M	FLOPs/G
YOLOv5s	87.7	74.2	81.4	64.1	7.02	15.8
YOLOv5m	89.4	76.8	83.7	48.3	20.9	47.9
M-YOLOv5s	85	76.6	82.1	63.6	3.73	9
M-YOLOv5m	89.8	76.7	84.1	53.8	11.3	29.3
ML-YOLOv5	86.1	77.7	82.8	63.6	3.73	9

**Table 6:** mAP comparison of the original model and the improved model.

Label Name	CPLID		IDID	
	YOLOv5	ML-YOLOv5	YOLOv5	ML-YOLOv5
strings	93.9	94.5	97.9	98.2
broken	99.5	99.5	70.3	74.8
flashover	\	\	73.2	74.5
hammer	\	\	84	83.4

## Data Availability

The data that support the findings of this study are available from the corresponding author upon reasonable request. The CPLID is available online at https://github.com/InsulatorData/InsulatorDataSet, (accessed on 1 September 2022). The IDID is available online at https://doi.org/10.21227/vkdw-x769 (accessed on 20 September 2022).
